# Supplementary material for: Uncovering the Model and Philosophy of Care of a Psychiatric Inpatient Mother-Baby Unit in a Qualitative Study with Staff
Source: Int J Environ Res Public Health. 2022 Aug 7;19(15):9717. doi: 10.3390/ijerph19159717 (PMC9367725; doi:10.3390/ijerph19159717)
Supplement: Supplementary file 1 [file ijerph-19-09717-s001.zip › ijerph-1811301-supplementary.pdf]

Supplementary Table S1. Summary of themes, sub-themes, codes and sub-codes.

Themes	Sub-Themes	Codes	Sub-Codes
Model of Care			
	Mental Health Care	Primary goal	
		Comprehensive perinatal mental health assessment	Psychosocial risk factors
		Knowledge of perinatal mental health disorders	
		Impact of maternal mental health on baby	
		Crisis	
		Interventions	Pharmacological<br>Psychosocial
	Physical Health Care	Physiotherapy assessment	Pregnancy<br>Post-birth care<br>Pelvic floor<br>Pain<br>Bowel and bladder<br>Exercise engagement<br>Musculoskeletal history
		Physiotherapy interventions	Exercise
		Diet	Cooking
		Sleep	
		Post-caesarean care	
		Impact of baby's issues on mother's health	
	Babies' Care	Child health issues	Baby sleep<br>Settling<br>Co-sleeping<br>Feeding
		Mother-crafting	Functional assessments of parenting occupations
		Deterioration in baby health	
		Baby's development	Baby play<br>Baby massage
		Child Safety	Reporting<br>Assessment<br>Support for those involved with Child Safety
	Building mother-baby relationship	Mother-baby as one	
		Difficulties with maternal-infant attachment	
		Improving maternal-infant attachment	
		Nursing interventions	
		Allied health interventions	
	Fostering relationship with supports	Family member relationships	
		Involvement through from care planning to discharge planning	
		Couple relationships	
		Adjustment to parenthood	
		Family time	
	Facilitating community support	Community supports post-discharge	
		Acute timeframe	
		Telehealth	
		Range of service	
		Involvement in care	
Philosophy of Care			
	Person-centred care		
	Trauma-informed care		
	Compassion-centred care		
	Recovery-oriented care		

Attachment-informed care
Non-judgemental care
Strengths-based care
Interdisciplinary care
